# Deficits in Behavioral Functions of Intact Barrel Cortex Following Lesions of Homotopic Contralateral Cortex

**DOI:** 10.3389/fnsys.2018.00057

**Published:** 2018-11-22

**Authors:** Rahul Chaudhary, V. Rema

**Affiliations:** National Brain Research Centre, Manesar, India

**Keywords:** somatosensory, gap-crossing behavior, plasticity, tactile sensation, brain injury, stroke, vibrissae, diaschisis

## Abstract

Focal unilateral injuries to the somatosensory whisker barrel cortex have been shown cause long-lasting deficits in the activity and experience-dependent plasticity of neurons in the intact contralateral barrel cortex. However, the long-term effect of these deficits on behavioral functions of the intact contralesional cortex is not clear. In this study, we used the “Gap-crossing task” a barrel cortex-dependent, whisker-sensitive, tactile behavior to test the hypothesis that unilateral lesions of the somatosensory cortex would affect behavioral functions of the intact somatosensory cortex and degrade the execution of a bilaterally learnt behavior. Adult rats were trained to perform the Gap-crossing task using whiskers on both sides of the face. The barrel cortex was then lesioned unilaterally by subpial aspiration. As observed in other studies, when rats used whiskers that directly projected to the lesioned hemisphere the performance of Gap-crossing was drastically compromised, perhaps due to direct effect of lesion. Significant and persistent deficits were present when the lesioned rats performed Gap-crossing task using whiskers that projected to the intact cortex. The deficits were specific to performance of the task at the highest levels of sensitivity. Comparable deficits were seen when normal, bilaterally trained, rats performed the Gap-crossing task with only the whiskers on one side of the face or when they used only two rows of whiskers (D row and E row) intact on both side of the face. These findings indicate that the prolonged impairment in execution of the learnt task by rats with unilateral lesions of somatosensory cortex could be because sensory inputs from one set of whiskers to the intact cortex is insufficient to provide adequate sensory information at higher thresholds of detection. Our data suggest that optimal performance of somatosensory behavior requires dynamic activity-driven interhemispheric interactions from the entire somatosensory inputs between homotopic areas of the cerebral cortex. These results imply that focal unilateral cortical injuries, including those in humans, are likely to have widespread bilateral effects on information processing including in intact areas of the cortex.

## Introduction

Tactile spatial acuity in humans, as well as non-human primates, is said to depend on activity in distributed neural network generated through “interaction between bottom up tactile inputs and top-down attentional signals” (Sathian, 2016). In order to perceive the roughness of an object humans (Hsiao et al., 1993; Hollins et al., 2006) and monkeys (Sinclair et al., 1996) scan the surface of the object with their fingers. However, the rodents explore an object by active movement of their large facial whiskers (vibrissae) across the surface and the subsequent sensorimotor interactions occurring in the whisker to barrel cortex system control various whisker-dependent behaviors (Feldmeyer et al., 2013).

Inputs to primary somatosensory cortex integrate bilaterally with continuous interactions between the somatosensory areas in both the hemispheres (see review Tamè et al., 2016). Hence while learning a somatosensory task, using bilateral somatosensory inputs, activity-dependent plastic changes in somatosensory areas of both hemispheres would be induced due to the reciprocal interhemispheric interactions. This implies that the performance of the task at the highest degree (maximum level) of ability would depend on active somatosensory inputs to both hemispheres. We hypothesized that unilateral lesions would disrupt these bilateral interactions. Such disruptions would affect the behavioral functions of the intact contralesional hemisphere due to occurrence of diaschisis (von Monakow, 1914; Carrera and Tononi, 2014), to alter performance of a behavior that was previously learnt using bilateral sensory inputs. The whisker-barrel pathway of the rat is an ideal model system for testing this hypothesis because of vast amount of knowledge available on somatosensory information processing in this system (see recent reviews by Lampl and Katz, 2017; Campagner et al., 2018; Estebanez et al., 2018; Yang et al., 2018).

Acquisition and processing of tactile information through the whisker-barrel pathway is complex. Several studies imply that complex intracortical and intercortical interactions are necessary for functions of the whisker pathway. During contact with an object the bending moment, axial force and lateral force of the whiskers produces mechanical forces that act on whisker follicles and hence different kinds of whisker explorations influence the mechanotransduction (Szwed et al., 2003, 2006; Pammer et al., 2013; Bush et al., 2016; Campagner et al., 2016). Neurons in the whisker-barrel pathway are known to selectively encode spatial and dynamic features of sensory stimuli with cortical neurons exhibiting selectivity to more complex and context-dependent collective whisker motion (Bale and Maravall, 2018). Similarly, multiwhisker and multidirectional stimulation of whiskers sharpen receptive fields of whiskers (Ramirez et al., 2014). Also, activation of whisker sensory barrel cortex using optogenetic technique was shown to elicit retraction of the contralateral whiskers and protraction of the ipsilateral whiskers (Auffret et al., 2018).

Long-lasting deficits in neuronal activity and use-dependent plasticity in the reciprocally connected intact regions of the contralesional hemisphere are seen in rats with unilateral barrel cortex lesions (Rema and Ebner, 2003). Similar lesions were shown to affect the dynamics of whisker movement (Harvey et al., 2001). The influence of these long-lasting neuronal changes on behavioral function of the intact cortex is not well understood. A clear understanding of lesion-induced functional deficits in intact cortical regions would depend on choice of a suitable behavior. A behavioral task that is acquired and learnt using bilateral inputs could therefore address the nature of deficits and mechanisms that cause such deficits in behavior. In the recent review, Stüttgen and Schwarz (2018) have listed the large repertoire of behaviors that have been assigned to the whiskers, including swimming, prey capture, social interactions, nipple attachment during nursing, texture and aperture discrimination and gap-crossing. However, according to them the barrel cortex might be not critical for many of these behavioral tasks. Hong et al. (2018) have shown that although optogenetics inactivation or lesioning the barrel cortex has transient effect on whisker mediated detection of a pole to release a lever with the forepaw for water reward the recovery of this behavior occurred in the absence of barrel cortex.

Hutson and Masterton (1986) demonstrated that intact barrel cortex is essential for the performance of the “Gap-crossing” task. However, their experiments have shown that barrel cortex is not needed for the whiskers to detect passive stimuli such as an air stream on the whisker or for the whiskers to discriminate different frequencies of air puff stimulation of the whiskers. The Gap-crossing task requires the rat to navigate a path along a platform and cross a gap and jump to a second platform after actively palpating the edge of the second platform with its whiskers. We therefore used Gap-crossing task for this study. We made unilateral injury to the somatosensory (barrel) cortex in rats that were trained on Gap-crossing task using bilateral somatosensory input. We then quantified the deficits in this sensory-guided behavior and estimated the level of recovery at various post-lesion times. In the present study, we addressed two questions: (i) What are the long-term effects of unilateral focal lesions in the somatosensory cortex on somatosensory behavior? (ii) What is the possible mechanism of injury-induced behavioral deficits?

## Materials and Methods

Thirty out-bred Long Evans rats (3–6 months) were used for these experiments. This study was carried out in accordance with the recommendations of “NIH guidelines”. The protocol was approved by the “Institutional Animal Ethical Committee of the National Brain Research Centre”. The rats, aged 3 months, were obtained from the National Brain Research Centre animal breeding facility, and were maintained on a 12 h light/12 h dark cycle with unrestricted access to food and water. All animals were behaviorally trained and tested during the active (dark) period of their daily cycle. Each rat was handled for 10 min per day for 10 days prior to the beginning of behavioral training. The handling was done to familiarize the rats with presence and touch of the experimenter in the room where behavioral training and testing was done, and to rule out stress-induced changes in behavior or effect of novel environment on learned behavior.

### Gap-Crossing Behavior

**Figure 1:**
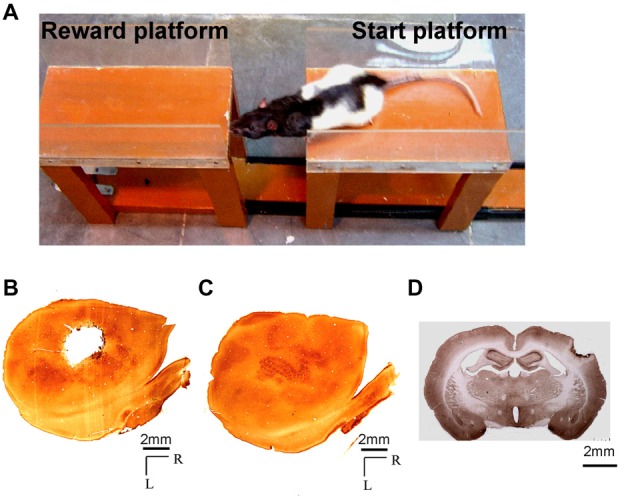
**(A)** Photograph showing a rat performing the Gap-crossing task. The rat jumps across a gap to the fixed “Reward-platform” from the “Start-platform” which can be moved to change the size of the gap. **(B,C)** Photomicrographs of cytochrome oxidase stained sections through layer 4 of the flattened cortex of **(B)** the lesioned hemisphere, and **(C)** the contralesional hemisphere showing a typical barrel cortex lesion encompassing the entire whisker representation (a.k.a. the posteromedial barrel subfield). **(D)** Coronal section from brain of a lesioned rat showing the depth of the lesion. Scale bar = 2 mm; R, rostral; L, lateral.

Cortically-dependent sensory function acquired by the mystacial vibrissae of rats was assessed using the Gap-crossing task (Hutson and Masterton, 1986). The Gap-cross apparatus, built in-house, was based on the description of Hutson and Masterton (1986). It consisted of two wooden platforms, each 30 cm long and 8 cm wide, with plexiglass side walls of 6 cm height. The platforms were raised above ground by 30 cm. One platform was mounted on a channel and was movable (Start-platform) and the other was fixed (Reward-platform). Hence, the gap between the platforms could be adjusted as desired (Figure 1A).

Acclimatization to the apparatus, training and behavioral testing were done in total darkness to eliminate any visual cues. The animals were acclimated to the Gap-cross apparatus by allowing them to explore the apparatus for 2 days with all of their whiskers intact and find the food reward placed on the Reward-platform. During acclimatization there was no gap between the platforms. Food was removed from the cage 12 h prior to acclimatization and training in order to motivate the animals to perform the task. The rats were trained to get the reward by crossing the gap separating the platforms. All the animals were trained on the apparatus with all their whiskers intact. A trial consisted of the rat moving from the Start-platform to the Reward-platform. Before starting each trial, the room lights were on and the platforms were cleaned with 70% alcohol. A single chocolate flavored cornflake placed in a 60 mm petridish attached to the far end of the Reward-platform was used as a reward for motivating the rat to jump across the gap. The rat was gently picked up from its cage. The room lights were turned off and the trials began when the rat was placed on the far end of the Start-platform. The rat then moves toward the gap and jumps across the gap to the Reward-platform. The trial ended as soon as the rat landed on the Reward-platform. For animals that did not cross the gap individual trials were terminated after a maximum time limit of 180 s (Barnéoud et al., 1991). After the trial ended the room lights were turned on, the rat was placed in its cage, the platforms were cleaned and food reward was placed for the next trial. During training, on the 1st day, the gap between the platforms was increased by 2 cm up to a distance of 10 cm. Subsequently the gap was increased at 1 cm increments until the rat was unable to contact the platform with its whiskers, and therefore refused to jump across the gap (for two rats the largest 2 cm gap was retested with 0.5 cms increments). On the following 2 days, the gaps between the platforms were increased and decreased randomly in order to prevent the rats from indiscriminately jumping across the gap but to induce the animals to make tactile contact with the Reward-platform with their whiskers prior to jumping to the Reward-platform.

We observed that when the platforms were separated by distances less than 11 cm the animal would touch the Reward-platform with its nose in addition to its whiskers, as reported by other investigators (Hutson and Masterton, 1986; Jenkinson and Glickstein, 2000). However, when the platforms were separated by larger gaps the rat could not reach far enough to touch the Reward-platform with its nose, and at that point they used their whiskers exclusively to detect and judge the distance before crossing. Video recordings taken during training confirmed that prior to crossing the widest (maximum) gap the animals touched the Reward-platform only with their whiskers. Five rats were excluded from these experiments. Three of these rats did not cross the gap even at small gap-widths that they could step across, but instead would attempt to climb down the Start-platform, one rat continued to leap across the gaps without contacting the Reward-platform throughout training, and one rat did not perform the task at criterion levels. The training lasted until the animals reached plateau performance (>85% correct) in crossing at the maximum distance. All rats included in this study reached plateau performance within 5–7 days. After the rats had attained criterion performance, their Gap-crossing ability was recorded for the next 2 days on video, using the infrared night vision setting, with Sony Handycam SR10E, at 30 frames per second, for offline analysis. The camera was adjusted to view the gap and edges of Start-platform and Reward-platform. We were able to see the contact of the whiskers on the reward platform but could not identify individual whiskers. We did not examine whether the rats modified their actual gap-crossing behavior after the lesion. The maximum gap-width crossed by a rat with all the whiskers intact on both sides was considered as “Control” or “pre-lesion” value for that animal. Subsequently rats were divided into following experimental groups.

#### Group 1 (Rats With Untrimmed Whiskers on Contralateral Side of Lesion)

The rats (*n* = 6) had barrel cortex lesion in the left hemisphere and the whiskers on the left side of the face were trimmed to level of fur, while whiskers on the right side were untrimmed. In these lesioned animals, since the intact whiskers projected to the lesioned hemisphere their Gap-cross performance provided information about the deficits in tactile behavior as a direct consequence of lesion.

For three of the rats (Group 1a) whiskers on the left side were trimmed. The animals were then tested on the Gap-crossing ability 1 h after whisker trimming and these data formed part of Group 4 experiments of Gap-crossing performance with unilateral whiskers (see below). Thereafter, cortical lesion was made in the hemisphere contralateral to the untrimmed whiskers. On post-lesion day (PLD) 7, the ability of these rats to perform Gap-crossing using contralesional whiskers was tested (data from one of the animals was not included because did not jump across the gap on PLD7). For the other three animals of this group (Group 1b) unilateral lesions were made in the barrel cortex. They were then tested on PLD7 with bilateral whiskers and these data formed part of Group 3 experiments to test the effect of lesion on Gap-crossing with bilateral whiskers (see below). The whiskers ipsilateral to the lesion were then trimmed and the rats were tested again for Gap-cross performance. Post-lesion Gap-cross behavior of all the six rats was similar.

#### Group 2 (Rats With Untrimmed Whiskers on Ipsilateral Side of Lesion)

This group of animals (*n* = 8) had unilateral barrel cortex lesion and were tested on Gap-crossing ability using whiskers that project to intact cortex. Prior to lesion, the whiskers on one side of the face were trimmed. One hour after whisker trimming they were tested for Gap-crossing behavior and these data formed part of the Group 4 experimental data that tested the Gap-crossing ability using unilateral whiskers (see below). Barrel cortex lesions were then made on the hemisphere ipsilateral to the untrimmed whiskers. Hence, for all these animals the intact hemisphere received sensory inputs from untrimmed whiskers. The Gap-cross performance of these animals was used to determine the ability of unilaterally intact whisker to cortex pathway to support the behavioral function.

#### Group 3 (Rats With Unilateral Lesion and Untrimmed Bilateral Whiskers)

In this group the rats (*n* = 7) had all the whiskers on both sides of the face, but the barrel cortex on the left hemisphere was lesioned. On PLD7 the Gap-crossing ability of these rats was tested. The Gap-crossing performance of these animals indicated whether use of the whiskers on both sides of the face had an effect on the amount of deficit. As mentioned above, data for three rats were obtained from Group 1b.

#### Group 4 (Unilateral Whiskers Intact)

The rats in this group (*n* = 12) had normal cortex (no lesion), but whiskers were trimmed on one side of the face. Prior to whisker trimming the rats were tested for control performance on Gap-cross task. Then whiskers either on the right or left side of the face were trimmed. The animals were retested on Gap-crossing task 1 h after whisker trimming. As mentioned above, the three animals from Group 1a and eight animals from Group 2 tested before cortical lesions formed part of this group.

#### Group 5 (D Row and E Row Whiskers Intact Bilaterally)

This group of rats (*n* = 5) did not have cortical lesion. They were trained to perform the Gap-cross task with all bilateral whiskers. All the whiskers in A, B and C rows on both sides of the face were then trimmed to the level of facial fur. These rats were tested 1 h after whisker trimming on the Gap-crossing task using the intact D and E row whiskers on both sides of the face.

### Whisker Trimming

The rat was gently picked up and held firmly in one hand while stroking its fur with the other hand to minimize struggling. Using a pair of fine scissors, all the whiskers on one side of the face or all whiskers of A, B and C rows from both sides of the face were cut down to the level of the fur. The rat was then returned to its home cage. The whiskers were trimmed every 2 days until end of experiment. We did not observe any adverse reaction to whisker trimming.

### Cortical Lesion

A unilateral lesion was made in the barrel cortex by subpial aspiration using procedures described in Rema and Ebner (2003). The rat was anesthetized with intraperitoneal injections of a mixture of ketamine (90 mg/kg) and xylazine (10 mg/kg). An incision was made in the skin of the head, along the midline. The skin and muscles were retracted from the one side of the skull. An opening of ~3 mm in diameter was made in the skull, to expose the barrel cortex (2 to 5 mm posterior to bregma and 4 to 7 mm lateral to the midline), and the bone flap was removed. Barrel cortex was removed by aspiration of the tissue below the pia, using a pulled Pasteur pipette attached to a mild vacuum suction (Figure 1B). Lesions were limited to the cortical gray matter and did not involve the underlying white matter (Figure 1D) as confirmed by histology. Gelfoam was placed on the lesion site, and after the bleeding had stopped the opening was closed by replacing the bone flap and securing it in place with dental cement. The skin margins were sutured together and an antibiotic cream (Neosporin) was applied topically. Sham lesions were made in three animals (all surgical procedures minus the aspiration lesion) as a control for the effects of surgery. The dura was left intact in the sham lesioned animals and no damage was done to the underlying cortical tissue. The animals were given an antibiotic (Enrocin 10%, 0.4 ml/kg) after surgery. Post-operative recovery occurred in the animal’s home cage. One day after the lesion the animals resumed normal feeding, grooming and locomotion, and exhibited movements of their vibrissae. Harvey et al. (2001) observed changes in amplitude of whisker movement following similar barrel cortex lesion in rats. Intracortical microstimulation of whisker barrel cortex in mice has been shown to cause retraction of whiskers in mice (Matyas et al., 2010). Hence in these experiments it is possible that unilateral lesions of barrel cortex had affected the whisker movements. However, we did not examine the retraction, protraction and amplitude of whisker movements of the animals used in this study.

### Histology

At the end of the experiments, the animals were deeply anesthetized with ketamine and xylazine and were transcardially perfused with phosphate buffered saline (PBS) followed by 4% paraformaldehyde in PBS. The brain was removed and cryoprotected by sequentially immersing it in 10%, 20% and 30% sucrose in PBS (Rema et al., 1998). The lesioned and the intact hemispheres were flattened and frozen sections of 60 μm thickness were cut on a sliding microtome. The free-floating sections were reacted for cytochrome oxidase (Wong-Riley and Welt, 1980) to estimate the extent of the lesion. Figures 1B,C shows cytochrome oxidase reacted sections through layer 4 of the flattened cortex from one of the experimental animals illustrating the extent of the lesion in barrel field (Figure 1B) and the intact contralesional hemisphere (Figure 1C). For some experiments the brains were cut coronally at 40 μm thickness. In Figure 1D the photograph of the section from one experimental animal shows the depth of the lesion. The lesion is in the cortical layers and there is no direct damage to underlying white matter.

### Data Analysis

Video recordings of the Gap-cross behavior were saved in AVI format for offline analyses. The video frames were examined manually by an investigator who was blind to the experimental conditions. At every increment of gap, the ability of the animal to contact the Reward-platform with whiskers or the nose was monitored. For each animal the maximum gap-width that was crossed, following contact of the Reward-platform exclusively with its whiskers, was measured before and after experimental treatment. Whenever the rat came to the edge of the start platform and extended its snout and whiskers towards the gap we considered it as one attempt to contact the reward platform. If the gaps are small the animals usually crossed the gap on the first attempt. When the gap is larger the animal might not cross on the first attempt but retreats back to the start platform. It then approaches the gap again to cross and this was considered as the second attempt. The number of times the rat approaches the gap before it crosses the gap or the number of times it approaches during the trial period of 180 s was considered as the total number of attempts in a trial. In order to determine whether the various experimental treatments would affect subtle features of tactile behavior we examined three parameters of the Gap-cross performance. These were: (i) total time the animal was on the Start-platform until it crossed the gap; (ii) time spent by the animal probing the gap; and (iii) the number of times the rat approached the gap to contact the Reward-platform in an attempt to cross the gap. These parameters were measured during the trials at three gap-widths i.e., Gap*M*, which is the maximum gap-width crossed by the lesioned rat or unilateral whisker trimmed rat or rat with all whiskers of A, B and C rows trimmed bilaterally; Gap*M−1*, which is 1 cm smaller than Gap*M*; and Gap*M+1* which is 1 cm larger than Gap*M*. Each rat was given three trials at GapM, Gap*M−1*, Gap*M+1*. If the animal was able to cross the gap at least in one of the three trials it was considered as a successful gap-cross performance. During control performance the animals jumped across Gap*M−1*, GapM and Gap*+1* successfully on all trials. After experimental treatment the animals jumped across Gap*M−1* in all three trials. At GapM some of the animals did not jump cross on all the three trials but crossed at least on one trial. At Gap*M+1* they were unable to jump across the gap for all the three trials. This indicated that with increasing gap-widths there is reduction in accuracy of rats to jump across the gap.

The “Control” Gap-cross performance for each animal was noted. Following unilateral barrel cortex lesion or whisker trimming, we compared the performance of the animal with its control performance. Individual animal data showing the maximum gap-width jumped at “Control” performance and after experimental treatment are presented in line graphs. For comparing the ongoing changes in performance at various post-lesion times, we normalized the pre-lesion performance to 100% for each rat. The data were averaged for all the animals subjected to same experimental treatment. The data are presented as mean (±SEM). The significance of differences in the behavioral performance of animals following an experimental treatment compared to “Control” performance was determined using Paired *t*-test (SigmaStat).

## Results

The Gap-crossing task, a whisker-dependent tactile behavioral task, was used to determine contralesional somatosensory deficits in animals with unilateral lesions of the barrel cortex. All rats used all the whiskers on both sides of their face during learning to perform the Gap-crossing task. The maximum gap-width jumped by each rat prior to experimental treatment was the “Control” Gap-cross performance for that rat. The experimental treatments were whisker-trimming and cortical lesions. After experimental treatment we measured the width of gap jumped across by each rat.

Additional parameters of the Gap-crossing behavior were also analyzed in detail. The parameters that we examined were: (i) the total time spent by the rat on the Start-platform prior to crossing the gap; (ii) time spent probing the gap; and (iii) the number of attempts made by the rat to cross the gap. These parameters were examined at three gap-widths i.e., Gap*M*, Gap*M−1* and Gap*M+1*.

Gap*M*: this is the maximum gap-width that each experimentally treated animal (after lesion or after whisker trimming) was able to jump across.

Gap*M−1*: this gap-width is 1 cm less than Gap*M*. At this gap-width the animals jumped across the gap with ease. At this gap-width we did not find significant differences in the three above mentioned parameters between lesioned/whisker trimmed and control performance.

Gap*M+1*: this gap is 1 cm more than Gap*M*. At Gap*M+1* the rats with either lesion or unilateral whisker trimming did not jump across the gap. However, prior to lesion or whisker trimming, the rats had successfully crossed this gap.

### Deficits in Tactile Behavior Using Whiskers Projecting to the Lesioned Barrel Cortex (Group 1 Rats)

**Figure 2:**
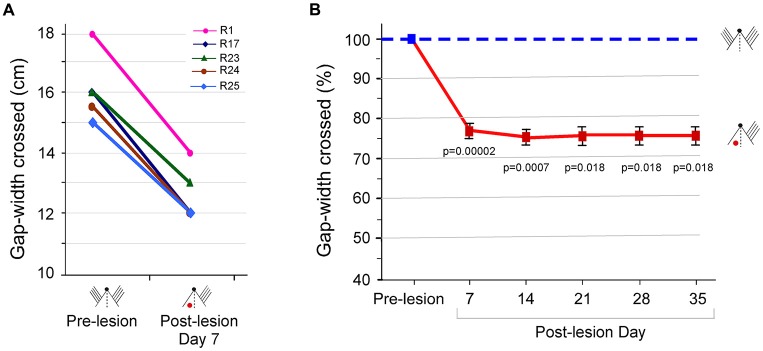
Performance at the gap-crossing task by rats with unilateral barrel cortex lesion using the whisker inputs to the lesioned cortex. **(A)** Maximum Gap-widths crossed by individual rats, R1, R17, R23, R24 and R25, at pre-lesion and on post-lesion day (PLD) 7 showing reduction in the gap-width crossed by rats after lesion. **(B)** Line graphs showing ongoing deficits in Gap-crossing performance of lesioned rats up to 5 weeks post-lesion (solid red line) compared to pre-lesion performance (blue dashed line). Squares are mean ± SEM, (pre-lesion = blue square, post-lesion = red squares). *p*-values are for pre-lesion vs. lesion performance in Paired *t*-test. Small figurines depict the experimental conditions on a cartoon of a rat head viewed from above. The black dot represents the nose, and the parallel lines represent the intact whiskers. The “red dot” indicates the location of the barrel cortex lesion.

The Gap-crossing task is a whisker-dependent tactile behavioral task for rats. Since intact somatosensory cortex has been shown to be essential for performance of the Gap-cross task (Hutson and Masterton, 1986), we first examined the direct consequence of unilateral somatosensory cortex lesion on somatosensory behavioral functions. Pre-lesion (Control) Gap-crossing behavior of six rats with all bilateral whiskers was determined. After barrel cortex lesion, we tested Gap-crossing performance of the rats on PLD7, using whiskers contralateral to the lesioned barrel cortex. The rats showed a reduction in the gap-width crossed when contact with the Reward-platform was restricted solely to the whiskers projecting to the lesioned cortex. Similar reduction in Gap-crossing performance of rats with unilateral barrel cortex lesion has been observed in other studies (Hutson and Masterton, 1986). They first trained the rats on the Gap-crossing task with one whisker on each side of the face and then lesioned the barrel cortex unilaterally. The gap-crossing ability of the rats was examined on post-lesion day 10 with the whisker projecting to the lesioned hemisphere. In our experiment as shown in Figure 2A, we found that the maximum gap-width crossed by the lesioned rats on PLD7 was 3–4 cm less than the control pre-lesion performance (one rat did not jump across the gap on PLD7 hence it was removed from the analyses). At these smaller gap-widths, we observed that the animals could contact the Reward-platform with their nose, as reported in other studies (Hutson and Masterton, 1986; Harris and Diamond, 2000; Jenkinson and Glickstein, 2000; Morita et al., 2011). This suggested that deficit in Gap-crossing behavior was specific to lack of whisker barrel cortex, while somatosensory information processing from other parts of body, such as the nose, is not affected.

We continued to monitor the Gap-crossing performance of the rats every week, for 5 weeks post-lesion. As seen in Figure 2B, the lesioned rats could cross only smaller gap-widths, and this deficit persisted for at least until 5 weeks post-lesion. The average reduction in the gap was 21.7 ± 1.08% at PLD7 (*P* = 0.00002, *n* = 5), 24.1 ± 0.9% at PLD14 (*P* = 0.0007, *n* = 3), 23.6 ± 1.38% at PLD21 (*P* = 0.018, *n* = 2), 23.6 ± 1.38% at PLD28 (*P* = 0.018, *n* = 2) and 23.6 ± 1.38% at PLD 35 (*P* = 0. 018, *n* = 2) compared to “Control” performance. Thus, lesion of barrel cortex that receives sensory input degrades successful performance of the Gap-crossing task with the whiskers.

### Whisker-Dependent Behavioral Function of the Intact Barrel Cortex in Rats With Unilateral Barrel Cortex Lesion and Untrimmed Ipsilesional Side Whiskers (Group 2 Rats)

**Figure 3:**
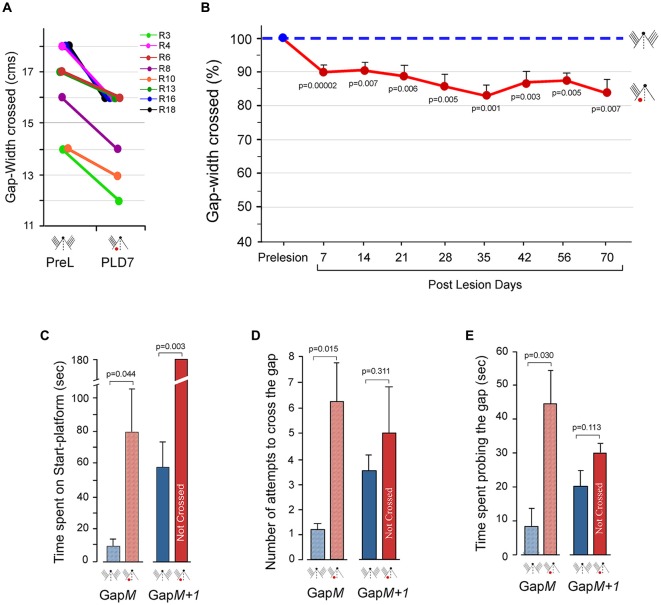
Performance at the Gap-crossing task by rats with unilateral barrel cortex lesion using unilateral whisker inputs to the intact cortex. **(A)** The maximum distance jumped by individual animals on PLD7 is compared to the maximum distance they jumped before the lesion. Following unilateral barrel cortex lesion all the eight animals could cross only smaller gap-widths using the whiskers that project to the intact cortex. R3, R4, R6, R8, R10, R13, R16, R18 are numbers for individual rats. PreL, pre-lesion; PLD, post-lesion day. **(B)** Line graphs showing long-lasting deficits in performance on the Gap-crossing task by the rats with unilateral barrel cortex lesion using whiskers projecting to intact barrel cortex. The reduction seen in Gap-crossing performance by PLD7 persists at all post-lesion times examined (red solid line) compared to pre-lesion performance (blue dashed line). Circles (pre-lesion = blue, post-lesion = red) are Mean ± SEM. **(C)** Total time spent on the Gap-cross apparatus by the rats (*n* = 4) during the trials at Gap*M* and Gap*M+1* gap-widths. Gap*M* is the maximum gap-width crossed by the lesioned rats. Gap*M+1* is 1 cm wider that Gap*M*. The rats spend significantly more time at Gap*M* after unilateral barrel cortex lesion (red hatched bar) compared to the time spent at pre-lesion (blue hatched bar). At Gap*M+1*, the lesioned rats (red solid bar) do not cross the gap until the termination of the trial but at pre-lesion they had crossed the gap (blue solid bar). **(D)** Number of approaches to the gap made by the rats (*n* = 4) in attempt to cross Gap*M* and Gap*M+1*. The number of attempts by lesioned rats is significantly more at Gap*M* (red hatched bar) than at pre-lesion (blue hatched bar). Whereas at Gap*M+1* there is no significant difference in the number of attempts made by the rats after lesion (red solid bar) compared to pre-lesion (blue solid bar). **(E)** Total time spent probing the gap at Gap*M* and Gap*M+1*. The rats (*n* = 4) spend significantly more time probing the gap at Gap*M* after lesion (red hatched bar) compared to pre-lesion (blue hatched bar), while at Gap*M+1*, there is no significant difference in time spent at the gap between lesion (red solid bar and pre-lesion performance (blue solid bar). *P* values were calculated using Paired *t*-test. Small figurines coded as in Figure 2.

Can the animals with unilateral barrel cortex lesions use sensory inputs from whiskers to the intact contralateral cortex to successfully perform the Gap-crossing task? To address this question, we examined the performance of rats with unilateral barrel cortex lesion (*n* = 8) using all whiskers ipsilateral to the lesion side, i.e., the whiskers that project to the intact hemisphere. “Control” Gap-cross performance behavior was determined, prior to cortical lesion, by measuring the maximum gap-width crossed by each rat using all whiskers on both sides of the face. All whiskers on one side of the face were then trimmed and barrel cortex ipsilateral to the trimmed whiskers was lesioned. On PLD7 the lesioned rats were tested on Gap-crossing task. The rats performed the task using the whiskers that projected to the intact barrel cortex. Although the rats could contact the Reward-platform with their whiskers, they did not cross the maximum distance that they had crossed previously before the lesion. Figure 3A shows the maximum gap-width crossed by individual rats in Group 2, before and after lesion. The average reduction of 9.9 ± 1.1% in the gap-width crossed by the lesioned rats is significant compared to their control performance (*P* = 0.00002).

### Persistence of Deficits in Somatosensory Behavioral Functions of Intact Contralesion Barrel Cortex

Single neuron studies have shown that spontaneous and evoked activity in the intact barrel cortex were persistently reduced up to 120 days following a lesion in the contralateral barrel cortex (Rema and Ebner, 2003). This suggests that the deficits in the behavioral functions of intact contralesional somatosensory cortex may not recover even at longer post-lesion times. Hence, after unilateral lesions of the barrel cortex the Gap-crossing ability of the rats (*n* = 8), using whiskers that project to intact barrel cortex, was examined at different post-lesion times up to PLD70 (one rat died on PLD66). Figure 3B shows the mean reduction in the gap-width crossed by lesioned rats (solid red line) compared to their pre-lesion (dashed blue line) performance, on PLD7 (9.1 ± 1.1%; *n* =8; *P* = 0.00002), PLD14 (9.6 ± 2.8%; *n* = 8; *P* = 0.007), PLD21 (11.9 ± 3.2%; *n* = 8; *P* = 0.006), PLD28 (14.0 ± 4%; *n* = 8; *P* = 0.005), PLD35 (17.0 ± 3.6%; *n* = 8; *P* = 0.001), PLD42 (14.2 ± 3.1%; *n* = 8; *P* = 0.003), PLD56 (12.5 ± 2.2%; *n* = 8; *P* = 0.001) and PLD70 (16.1 ± 4.3%; *n* = 7; *P* = 0.007). The maximum gap-width jumped by each rat prior to lesion was considered as 100% performance (Figure 3B dashed blue line). The reduction in the maximum gap-width jumped at all post-lesion times remained 10%–18% less than the pre-lesion distance with no improvement up to PLD70, the longest time studied. This reduction is due to inability of the lesioned rats to jump larger gaps even though they were able contact the Reward-platform with their whiskers. These results indicate that focal unilateral lesions of cortex produce long-lasting impairments of behavioral capacity of the intact contralesional hemisphere.

We observed that the lesioned rats could jump across smaller gap-widths successfully after they contacted the Reward-platform indicating that there is no deficiency in judging the gap. However, at larger gap-widths although the rats could contact the edge of the Reward-platform with the distal ends of their whiskers they were unable to jump across the gap. This indicated that there were impairments in sensory processing of tactile information in the intact contralesional cortex when the task became difficult. Reduction in neuronal activity in the contralesional hemisphere following unilateral lesions in the barrel cortex, as reported by Rema and Ebner (2003), perhaps affects the performance of tactile behavior. This could influence the behavioral strategy used by the animal when the task becomes difficult. Therefore, on PLD7, we examined additional parameters of the Gap-cross performance of the lesioned rats (*n* = 4). The total time spent by the lesioned rats on the Start-platform until it initiated the jump, the number of times the animal approached the gap to probe the Reward-platform, and the time spend probing the gap were determined.

Lesioned rats jumped across easily at Gap*M−1* (which is 1 cm smaller than Gap*M*). Analyses of the total time spent on the Start-platform, number of attempts to cross the gap and the total time spent probing the gap showed that the performance of lesioned rats was similar to the pre-lesion performance. Interestingly, at Gap*M*, (the maximum gap-width that the lesioned rats could cross) there were significant differences in these parameters. As shown in Figure 3C (hatched bars), the lesioned rats spent longer time on the Start-platform compared to pre-lesion (PLD7 = 79.8 ± 25.7 s vs. Pre-lesion = 8.9 ± 4.9 s; *P* = 0.044). During this time period, the lesioned animals made more approaches towards the gap in attempt to contact the Reward-platform (Figure 3D, hatched bars, Pre-lesio hatched bars, Pre-lesion = 1.25 ± 0.2 vs. PLD7 = 6.25 ± 1.5; *P* = 0.015) and also spent more time probing the gap with their whiskers compared to pre-lesion (Figure 3E, hatched bars, Pre-lesion = 7.9 ± 5.2 s vs. PLD7 = 44.88 ± 9.7 s; *P* = 0.030).

On increasing the gap by 1 cm (Gap*M+1*), although the lesioned animals could contact the Reward-platform with their whiskers they did not cross the gap in duration of the trial (180 s; Figure 3C, solid bars). However, the rats had crossed this gap-width in 58.6 ± 18 s prior to cortical lesion. There was no significant difference in the number of attempts to contact the Reward-platform (Figure 3D, solid bars; Pre-lesion = 3.75 ± 0.6 vs. PLD7 = 5 ± 2; *P* = 0.311) and in the time spent probing the gap (Figure 3E, solid bars; Pre-lesion = 20.8 ± 5 s vs. PLD7 = 30.1 ± 2.1 s; *P* = 0.1130).

When the gap between the platforms was reduced to 4 cm less than the maximum gap-width that the rats had crossed at pre-lesion, we observed that the lesioned rats touched the Reward-platform with their nose and jumped the gap with ease. At this nose-contact gap, the performance of the lesioned rats was no different than their pre-lesion performance. These results suggested that there was no decrement in the ability to carry out the task when the animals could get sensory inputs from the nose. Thus, the reduction in the maximum distance jumped by the lesioned rats is because the intact unilateral cortex is unable to support normal behavior.

### Deficits in Gap-Crossing Performance of Lesioned Rats Using Whiskers on Both Sides of Snout (Group 3 Rats)

As described above, rats with unilateral lesions of the barrel cortex exhibited persistent deficits in Gap-crossing despite contacting the Reward-platform with the whiskers that projected to intact barrel cortex. However, given that there is complex anatomical connectivity between subcortical whisker areas and other regions in the brain (Ahissar and Assa, 2016; McElvain et al., 2018), it is possible that the use of whiskers on both sides of the face for Gap-crossing could result in better behavioral performance in animals with unilateral barrel cortex lesion. Also, de Lafuente and Romo (2006) have shown that in response to sensory stimuli, there are progressive increases in the strength of covariations between neuronal activity and perceptual judgments across cortical areas, when the activity is transmitted from primary somatosensory cortex to the various premotor areas in the frontal cortex. This suggests that the active inputs from intact whisker to barrel pathway could have influence on the motor areas of both hemispheres and could affect Gap-crossing behavior.

**Figure 4:**
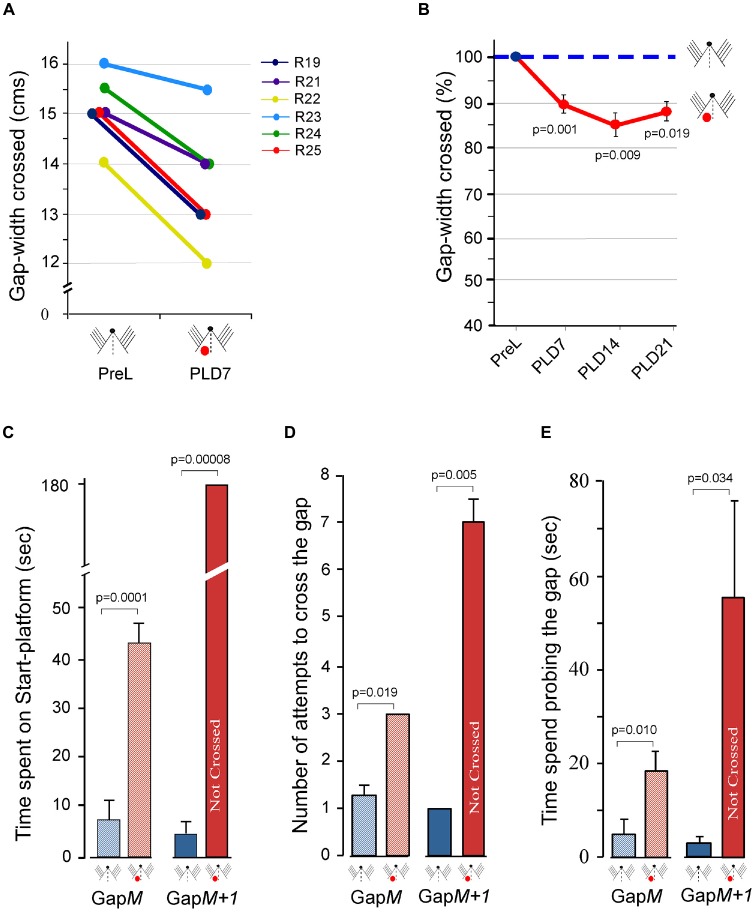
Effect of unilateral barrel cortex lesion on performance of Gap-crossing task by rats using all whiskers on both sides of the face. **(A)** Following unilateral lesions, the rats (R19, R21, R22, R23, R24, R25) jumped across smaller gap-widths on PLD7, using whiskers on both side of the face. **(B)** This significant reduction in the gap-widths that lesioned rats crossed seems to persist at longer post-lesion times. Circles (pre-lesion = blue; post-lesion = red) are Mean ± SEM. **(C)** Compared to pre-lesion behavior (blue hatched bar) the rats spend significantly more time at Gap*M* after unilateral barrel cortex lesion (red hatched bar). At Gap*M+1* the lesioned rats (red solid bar) are unable to cross the gap that they had crossed at pre-lesion (blue solid bar). **(D)** The number of attempts by lesioned rats (red hatched bar) compared to pre-lesion performance (blue hatched bar) is significantly more at *GapM* and at Gap*M+1* (pre-lesion = blue solid bar; post-lesion = red solid bar). **(E)** The rats spend significantly more time probing the gap after lesion compared to pre-lesion at Gap*M* (pre-lesion = blue hatched bar; post-lesion = red hatched bar) and at Gap*M+1* (pre-lesion = blue solid bar; post-lesion = red solid bar). *P* values were calculated using Paired *t*-test; PreL, pre-lesion; PLD, post-lesion day. Small figurines coded as in Figure 2.

We therefore, determined the maximum gap-width crossed by rats (*n* = 7) with unilateral barrel cortex lesion on PLD7, using all the whiskers on both sides. All the seven rats were able to jump across smaller gap-widths compared to their pre-lesion performance. Six rats could jump across gaps when gap-width was reduced by 0.5–2 cm. Since one rat could jump across only when the gap-width was reduced by 9 cm we did not include it in further analyses. Figure 4A shows the Gap-cross performance of the six rats in Group 3 at pre-lesion and on PLD7. The average reduction in the gap-width crossed by the lesioned rats on PLD7 using bilateral whiskers was 10.7% less than their pre-lesion performance (*P* = 0.001). We wanted to see if there is any recovery at longer post-lesion times. Hence, we continued to monitor the Gap-crossing ability of the lesioned rats on PLDs 14 (*n* = 4) and 21 (*n* = 3). As shown in Figure 4B, the average performance of the lesioned rats remained lower than their pre-lesion performance on PLD7 (10.1 ± 1.8%; *P* = 0.001), on PLD14 (14.8 ± 2.9%; *P* = 0.009) and on PLD21 (11.8 ± 2.1%; *P* = 0.019).

To further evaluate the behavioral deficits in this group of animals, the gap-crossing performance was examined in detail on PLD7 at Gap*M* and at Gap*M+1*. At Gap*M* the total time spend by rats on the Start-platform prior to initiating the jump was significantly more than pre-lesion (Figure 4C, hatched bars; PLD7 = 43 ± 5.5 s vs. Control = 7 ± 3.6 s; *P* = 0.0001). They showed significant increases in the number of attempts to cross the gap (Figure 4D, hatched bars; PLD7 = 3.0 ± 0.3 vs. Control = 1.3; *P* = 0.019) and the total amount of time spent on probing the gap to contact the Reward-platform (Figure 4E, hatched bars, Control = 4.0 ± 3.2 vs. PLD7 = 18.7 ± 4.3, *P* = 0.010).

On examining the Gap-crossing performance at Gap*M +* 1, we observed that prior to lesion the rats jump across the gap-width within 4.7 ± 2.2 s but on PLD7 the lesioned rats do not cross the gap-width until termination of the trials at 180 s (Figure 4C solid bars; *P* = 0.00008). These rats made significantly more attempts to cross the gap (Figure 4D solid bars PLD7 = 7.0 ± 0.6 vs. pre-lesion = 1 ± 0.0; *P* = 0.005) and spent significantly longer time probing the gap (Figure 4E solid bars, PLD7 = 56.7 ± 21.8 s vs. pre-lesion = 2.7 ± 1.2 s; *P* = 0.034).

The reduction in the gap-width crossed by the Group 3 rats is similar to that seen in Group 2 animals that had unilateral barrel cortex lesion and performed the task using whiskers that projected to the intact cortex (see Figure 3B). Results from these two groups of rats suggest that interactions from other intact regions in the brain do not compensate for the sensory deficits. These results also imply that the requirement of interhemispheric interactions between the barrel cortices is essential for optimal performance at the Gap-crossing task.

### Unilateral Whisker Removal Alone Affects Gap-Cross Performance of Normal Rats (Group 4 Rats)

The results presented above show that the animals with unilateral barrel cortex lesion had deficits in Gap-crossing behavior whether they used their whiskers providing input to the lesioned hemisphere or to the intact hemisphere or when both sets of whiskers were used. It is known that reciprocal interhemispheric connections are involved in processing sensory information necessary for normal behavior. We proposed that the behavioral dysfunction seen in the animals with unilateral barrel cortex lesion, specifically in the functions of intact hemisphere is likely to be caused by reduction in sensory inputs to the intact hemisphere rather than due to a generalized effect of the lesion. If this is true, then we should see a similar deficit if the sensory activity to one hemisphere is reduced by other means.

**Figure 5:**
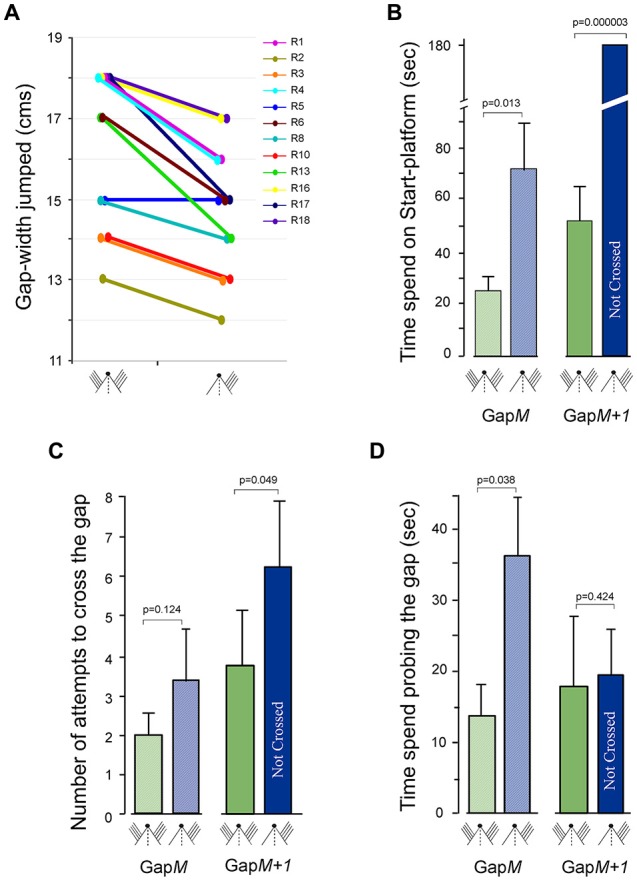
Gap-crossing behavior of normal rats following removal of whiskers on one side of face. **(A)** Line graphs showing the comparison of Gap-crossing task performance of each rat (R1, R2, R3, R4, R5, R6, R8, R10, R13, R16, R17, R18) using bilateral whiskers and unilateral whiskers. Compared to performance with bilateral whiskers the rats (*n* = 11; 91.6%) crossed smaller gap-widths after whiskers on one side of the face were trimmed. **(B)** At Gap*M* the rats spend significantly more time after unilateral whisker trimming (blue hatched bar) compared to all whisker intact bilaterally (green hatched bar) and at Gap*M+1* the rats are unable to cross the gap using unilateral whiskers (blue solid bar) that they had previously crossed with bilateral whiskers (green solid bars). **(C)** The number of attempts made by the rats to cross Gap*M* (hatched bars, green = bilateral whiskers, blue = unilateral whiskers) and Gap*M+1* (solid bars, green = bilateral whiskers, blue = unilateral whiskers) with unilateral intact whiskers is not significantly different compared to the attempts made with bilateral whiskers. **(D)** However, the rats spent significantly more time probing the gap at Gap*M* after unilateral whisker trimming (blue hatched bar) compared to bilateral control performance (green hatched bar), while at Gap*M+1*, there is no significant difference in time spent at the gap (solid bars, green = bilateral whiskers, blue = unilateral whiskers). *P* values were calculated using Paired *t*-test. Small figurines coded as in Figure 2.

Hence, we examined whether there are deficits in the Gap-crossing behavior when sensory inputs via whiskers are removed from one side in otherwise normal animals. We trained and recorded the normal Gap-crossing behavior of 12 rats. We then trimmed all whiskers on the one side of face for these animals and after 1 h we examined their Gap-crossing ability. As seen from the individual performance of the rats in Figure 5A, whisker trimming affected the gap-crossing ability of 11 (91.7%) rats. After whisker trimming they could only jump across gap-widths that were smaller compared to the gap-widths crossed with intact bilateral whiskers. This reduction of 10 (±1.8)% is despite the ability of the rats to contact the Reward-platform with untrimmed whiskers.

The total time spent on the Start-platform, number of approaches to the gap and the total time spent probing the gap was examined during Gap-crossing at Gap*M* and at Gap*M+1*. During Gap-crossing at Gap*M*, we observed that the rats with unilateral whiskers remained on the Start-platform for significantly longer time (74 ± 20 s) compared to their “Control” performance (21.3 ± 5.9 s; *P* = 0.013, Figure 5B hatched bars). Although the number of approaches to the gap by the rats using unilateral whiskers (2.0 ± 0.5) was not significantly different from their performance with bilateral whiskers (3.3 ± 1.4; *P* = 0.124; Figure 5C hatched bars), the rats spent significantly more time probing the gap (unilateral whiskers = 36.3 ± 8 s vs. bilateral whiskers = 17.8 ± 4 s *P* = 0.01; Figure 5D hatched bars).

At Gap*M+1* the rats crossed the gap in 52 ± 14.9 s using bilateral whiskers but did not cross the gap with unilateral whiskers until the end of trial (180 s; Figure 5B, solid bars). During this time there was no significant difference in the number of attempts to cross the gap (Figure 5C, solid bars), as well as in the time spent probing the gap (Figure 5D, solid bars) by the rats using unilateral whiskers compared to using bilateral whiskers.

### Bilateral Removal of Three Rows of Whiskers Affects Gap-Cross Performance of Normal Rats (Group 5 Rats)

**Figure 6:**
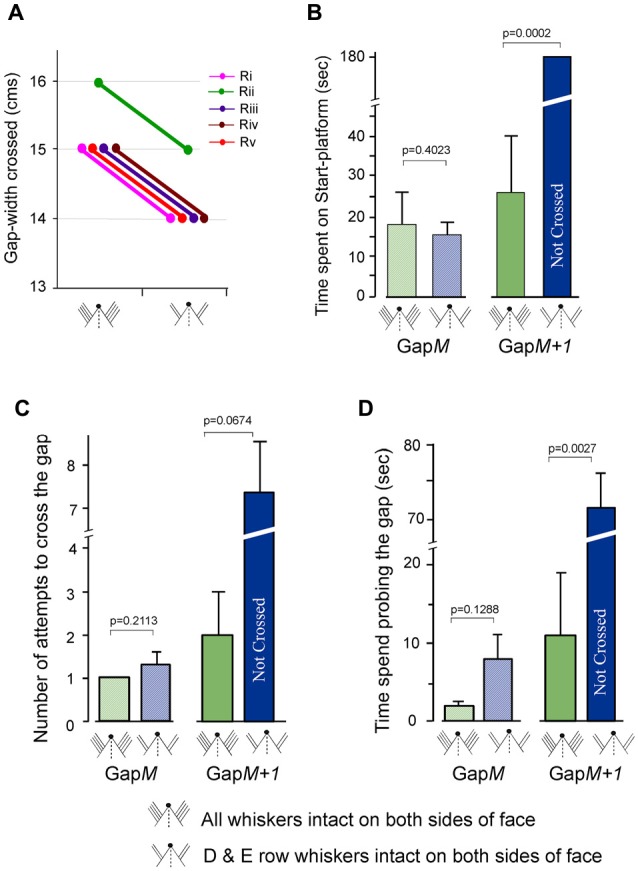
Gap-crossing behavior of normal rats following removal of A row, B row and C row whiskers from both sides of face. **(A)** Line graphs showing the comparison of individual performance on Gap-crossing task of five rats (Ri, Rii, Riii, Riv, Rv) using all whiskers intact bilaterally and with two rows (D row and E row) of whiskers intact bilaterally. Following whisker trimming all five the rats showed 1 cm reduction in the gap-widths they could cross at control performance. **(B)** At Gap*M* there is no significant difference in the total amount of time spent by the rats following whisker trimming (blue hatched bar) compared to all whiskers intact bilaterally (green hatched bar). Gap*M+1* the rats are unable to cross the gap using two rows of whiskers intact (blue solid bar) that they had previously crossed with bilateral whiskers (green solid bar). **(C)** The number of attempts made by the rats to cross Gap*M* (green hatched bar = all whiskers intact; blue hatched bar = D row and E row whiskers intact bilaterally) and Gap*M+1* (green solid bar = all whiskers intact; blue solid bar = D row and E row whiskers intact bilaterally) with two rows of intact whiskers is not significantly different compared to the attempts made with bilateral whiskers. **(D)** The time spent probing the gap at Gap*M* after bilateral whisker trimming A row, B row and C row (blue hatched bar) compared to control performance with all whiskers intact bilaterally (green hatched bar) is not significantly different, while at Gap*M+1*, there is significant difference in time spent at the gap after whisker trimming (blue solid bar) compared to control performance with all whiskers intact (green solid bar). *P* values were calculated using Paired *t*-test.

One possible cause for the deficits in performance of Gap-crossing behavior using whiskers projecting to the intact contralesional barrel cortex is that the sensory input from all whiskers that project to the intact cortex does not provide sufficient activity for sensory processing of tactile information when the rat contacts the platform across large gap-widths. It is also possible that the deficits are due to lack of bilateral interaction resulting from unilateral lesion. To test these two possibilities, we trained five rats on gap-crossing task with all the whiskers. We then trimmed the whiskers from rows A, B and C from both sides of the face. The performance of these rats, with D and E row whiskers intact bilaterally, on Gap-crossing was tested 1 h after whisker trimming. All five rats could cross smaller gap-widths of 1 cm less with two rows of whiskers intact on both side of face compared to the maximum gap-width they crossed prior to whisker trimming (Figure 6A). The reduction of 6.6 ± 0.8% is highly significant (*p* = 7.7E-08).

For three of the rats we examined the total time spent on the Start-platform, number of approaches to the gap and the total time spent probing the gap during Gap-crossing at Gap*M* and at Gap*M+1*. At Gap*M* the rats with two intact rows of whiskers did not have any significant difference in the three above mentioned parameters compared to control performance with all whiskers. During Gap-crossing at Gap*M* gap-width the rats with all whiskers spent an average of 18.2 ± 8.5 s on the Start-platform compared to 15.4 ± 3.2 s when using two rows of whiskers intact on each side of face (Figure 6B hatched bars, *p* = 0.40). The number of attempts was similar with all whiskers intact (1 ± 0.0) or after trimming three rows of whiskers bilaterally (1.3 ± 0.3; *P* = 0.211, Figure 6C hatched bars). Although the rats with bilateral D and E rows of whiskers spent more time at the gap (8.2 ± 3.9 s) than when all whiskers were used (2.3 ± 0.6 s) the difference was not significant (Figure 6C hatched bars, *P* = 0.128).

At Gap*M+1* the total time spent on the Start-platform (Figure 6B solid bars) was 25.8 ± 13.9 s when all whiskers are intact. After trimming A, B and C row whiskers bilaterally the rats could not cross the gap-width of Gap*M+1* during the trial period of 180 s (*P* = 0.00017). Although there is an increase in the number of attempts (Figure 6C solid bars) after whisker trimming (7.3 ± 1.2) compared to when all whiskers were intact (2.0 ± 1.0) it was not significantly different (*P* = 0.067). However, we saw that rats with two rows of bilaterally intact whiskers spent significantly more time probing the gap (71.5 ± 4.2 s) compared to when using all whiskers (11.2 ± 8.2 s, *P* = 0.0027, Figure 6D solid bars).

This result indicates that performance of Gap-crossing is affected when there is reduction of whisker inputs to both hemispheres. However in this group of rats since both hemispheres are receiving inputs from D and E rows of whiskers, there should be bilateral interaction between the barrel cortex areas of D and E rows of whiskers. This amount of interaction could be the reason that there is no difference at Gap*M* gap-width in the total time spent on the Start-platform, number of attempts and the time spent probing the gap by rats with two rows of whiskers intact compared to their control performance with all whiskers intact. But the fact that there are deficits in the performance at Gap*M+1* suggests that the amount of bilateral inputs from just two rows of whiskers is not enough for successful performance at larger gap-widths. It is possible that if more whiskers were intact bilaterally the deficits in Gap-crossing would be abolished.

These data imply that for peak performance of a bilaterally acquired somatosensory behavior a specific level of activity in each hemisphere is essential. This level of activity is achieved perhaps by a combination of direct input from all the peripheral somatosensory receptors to the sensory cortex and the interhemispheric transfer of information from the contralateral sensory cortex. These results reiterate that the deficits in behavioral functions of intact contralesional hemisphere are perhaps caused by loss of active inputs from the lesioned hemisphere and not because of generalized reaction to the cortical injury.

## Discussion

**Figure 7:**
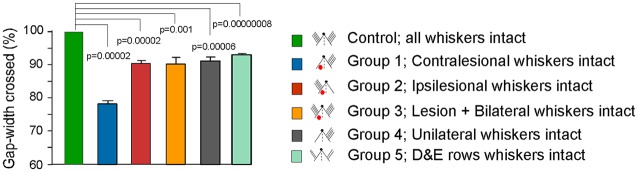
Summary figure showing Gap-crossing performance in controls and various experimental groups. Bar graphs comparing the gap-width crossed by different experimental groups with the “Control” performance. “Control” is the gap-width crossed by each animal with whiskers intact on both sides of the face prior to experimental manipulation and is normalized to 100% (see “Materials and Methods” section). For Groups 1, 2 and 3 Gap-cross performance at PLD7 is shown. For Group 4 performance with unilateral whiskers is shown and for Group 5 performance of rats with D and E row whiskers intact on both sides of face is shown. All animals in Groups 1, 2, 3, 4 and 5 show a significant reduction in the performance of Gap-crossing task compared to “Control” performance. Group 1 shows the greatest effect because the whisker inputs project to the lesioned cortex.

Our main findings, summarized in Figure 7, are that focal unilateral lesions in the somatosensory cortex in rats result in long-term deficits in the behavioral functions of the intact contralesional somatosensory cortex. Our experiments suggest that the impairments in the execution of a previously acquired behavior were specifically due to loss of active interactions between hemispheres. The results imply that learning a tactile behavior modifies the functions of each somatosensory cortex such that the subsequent performance of the learnt behavior at optimal level (maximum ability) depends on active bilateral interactions of somatosensory inputs in the somatosensory cortex. Therefore, if there is long-term dysfunction in tactile behavior following unilateral cortical injury, it could be due to a loss of unilateral somatosensory function or lack of insufficient sensory inputs from the periphery. The “Gap-crossing task” that we used to examine the somatosensory behavior in adult rats (Hutson and Masterton, 1986) requires the rat to contact a platform with its whiskers and use this tactile information for making decision about whether it is safe to jump across a gap of certain width for a reward. For performing this task intact barrel cortex is essential. Hence many studies have used the Gap-crossing behavior to test cortical functions (Sachdev et al., 2000; Lee et al., 2009; Chaudhary et al., 2013; Chu et al., 2013; Papaioannou et al., 2013; Wellmann and Mooney, 2015; Juczewski et al., 2016; Soumiya et al., 2016; Tsytsarev et al., 2017).

Hutson and Masterton (1986) showed that it is possible to train rats to perform the Gap-crossing task with a single whisker and others have shown this skill in mice (Celikel and Sakmann, 2007; Arnett et al., 2014). However, the rats used in our study had learned the task with all whiskers on both sides of the face. Following unilateral lesions in the barrel cortex, we found that the rats exhibited persistent deficits in the performance in Gap-crossing even when they used whiskers that project to the contralateral intact cortex. The characteristic feature of the deficit was the inability of rats to perform the Gap-crossing task even though the gaps were only wide enough that whiskers could still contact the Reward-platform. The deficits were greatest, however, when the animals were required to use only the whiskers that projected to the lesioned hemisphere. Our observation that rats are not able to cross the gap using whiskers that project to the lesioned cortex is similar to that shown in other studies in rats. Rats trained with bilaterally intact C1 whiskers showed impaired Gap-crossing when they had to use the C1 whisker projecting to lesioned hemisphere 10 days after lesion (Hutson and Masterton, 1986). In the study by Jenkinson and Glickstein, 2000) rats trained to cross the gap with all the whiskers showed reduction in the maximum gap that they could cross following disruption of connections from barrel cortex to pons by unilateral lesions of basis pedunculi when they used the whiskers that projected to lesioned region. Prior to testing with unilateral whiskers, they retrained the lesioned rats on the gap-crossing task using bilateral whiskers until they performed at prelesion levels. Whiskers were then trimmed unilaterally and animals were tested on the Gap-crossing behavior using whiskers that project to the lesioned pedunculi. Their results indicated that in addition to barrel cortex, cerebellar input is also important in the performance of this behaviour. In contrary to our results (Jenkinson and Glickstein, 2000) show that unilateral whisker trimming of normal trained animals did not reduce the maximum gap-width the animals could cross. The main difference between their experiment and ours is in the increment of gap-width for each successively larger gap that the rats had to cross. In their experiments the gap was increased by 2 cm whereas we increased the gap-width by 1 cm (0.5 cm in two cases). The gradual increase in the Gap-width perhaps is more sensitive in measuring deficits in the Gap-crossing behavior. In mice with unilateral lesion of barrel cortex, Barnéoud et al. (1991) saw deficits in gap-crossing at 2 week post-lesion and a partial recovery at 10 weeks when the whiskers contralateral to lesion were used. Hoffman et al. (2003) have shown that rats with unilateral cortical ischemia are significantly compromised in their exploratory behavior and on a two-texture discrimination task.

Long-lasting functional deficits in performing a series of behavioral tasks have also been shown in rats with unilateral middle cerebral artery occlusion, at least up to 90 days post-lesion (Lindner et al., 2003). The severity of the impairments showed a direct correlation with the volume of damaged tissue (Lindner et al., 2003). However, recovery of whisker mediated somatosensory function has been reported 56 days after unilateral photothrombotic ischemic lesions barrel field (Jablonka et al., 2010). The difference in the results of the above studies could be due to differences in experimental methodology such as the extent of damage to the brain, as well as the protocols used for assessing recovery. In our experiments we targeted the cortical lesions to encompass only the whisker barrel cortex. It is likely that over long post-lesion period additional ongoing reactions could have increased the lesion size as seen by Kozlowski et al. (1996) and Leasure and Schallert (2004). This enlargement of lesion size could include regions surrounding the barrel cortex such as secondary somatosensory cortex (S2). Also, Yamashita et al. (2018) have described that neurons in layers 2/3 of primary whisker barrel cortex have long range projections to whisker S2 (wS2) and also to primary and secondary whisker motor cortex (wMC). So unilateral lesions could also affect wS2 and wMC, which in turn would affect the behavior. In addition, unilateral barrel cortex lesion could alter somatosensory processing in the striatum. Reig and Silberberg (2014) used whole-cell recording and measured synaptic responses of striatal neurons to tactile stimuli delivered as brief air puffs to the whisker pads. They showed that neurons in the dorsolateral striatum responded to ipsilateral, contralateral as well as bilateral stimulation, with larger amplitude of response produced by bilateral stimulation. In their later study Reig and Silberberg (2016) showed that the although responses of striatal neurons to both ipsilateral and contralateral whisker stimulation are mediated through ipsilateral corticostriatal projections from primary somatosensory cortex (S1), the response to ipsilateral whiskers are through callosal inputs from contralateral barrel cortex suggesting interhemispheric interactions and integration.

In the present study, we show that impairments in behavior regulated by the intact somatic sensory cortex persisted in animals tested for up to 70 days after the lesion. Even though the whiskers could contact the Reward-platform, the failure of the lesioned rats in the “ipsilesional whiskers intact” Group 2 (Figure 7) to cross the gap indicates that the sensory information conveyed by the whiskers to the intact cortex was not being processed normally. Importantly, reducing sensory input by trimming all whiskers on one side of the face in a normal rat, (Group 4), produced similar reductions in Gap-crossing performance suggesting that execution of a previously acquired tactile skill requires the same inputs to optimally perform the task. This idea is reflected in the results of Harris and Diamond (2000). In their study, they trained the rats to perform Gap-crossing using a set of four unilateral whiskers. When the rats were tested after cutting those whiskers and gluing them to stumps on the opposite side of face corresponding to the position of the trained whiskers the rats exhibited reduced efficiency in performance of Gap-crossing task. This idea is further strengthened from our results that show similar deficits in behavior of animals in other groups (Figure 7). The animals of Group 2 have an intact input pathway to one hemisphere co-existent with a lesion in contralateral SI. The animals of Group 3 have a unilateral lesion in the barrel cortex and peripheral inputs to both hemispheres. In animals of Group 4 there was a decrease in the peripheral sensory inputs from the whiskers due to unilateral whisker trimming. While animals in Group 5 had two rows of whiskers intact on both sides of the face.

One of the goals of this study was to measure the extent of deficits in behavioral functions of the intact cortex. Using whisker input to the intact cortex, the Group 2 animals could cross the gap only when the gap-width was reduced by ~10% of the gap-width they crossed at pre-lesion. Comparable level of reduction is seen in Group 3, 4 and 5 animals (Figure 7). These results suggest that the Group 2, 3, 4 and 5 rats have inability to make a correct judgment of the distance even though they contacted the platform with whiskers projecting to the intact barrel cortex. Measurements of the three additional parameters of the Gap-cross behavior: (i) the total time the rats remained on the Start-platform; (ii) the number of approaches they made to the edge of the gap; and (iii) the total time spent probing the gap, also imply deficits in acquisition or processing of tactile information. At smaller gap-width i.e., Gap*M−1*, there was no significant difference in these parameters for Group 2, 3, 4 and 5 rats compared to their “Control” performance. However, when the gap-width was increased by 1 cm i.e., to Gap*M*, the time spent on the Start-platform, the number of attempts to cross the gap, and the amount of time probing the gap by rats of Groups 2, 3 and 4 were significantly more compared to their control performance. Whereas there was no significant difference in these parameters in rats with D and E rows whiskers intact bilaterally (Group 5) compared to their control performance suggesting that perhaps the bilateral interaction between areas of the two rows of whiskers influences the Gap-crossing behavior. With further increase of gap-width by 1 cm, i.e., at Gap*M+1*, the rats in Groups 2, 3, 4 and 5 could not jump across the gap, which they had crossed during control performance.

Therefore, the deficiencies in bilateral processing of sensory information within the barrel cortex occur when the interhemispheric processing of sensory inputs from the periphery are altered due to lesioning the cortex or due to trimming the whiskers. Since the deficiencies in behavioral ability in animals using unilateral whiskers were similar to those found in the lesioned animals, we conclude that they both disrupt bilateral processing of sensory information.

### Possible Mechanisms for Lesion-Induced Behavioral Deficits

#### Object Location Along the Whisker Shaft

During Gap-crossing the rat stretches across the gap to contact the Reward-platform with its whisker. At the maximum gap-width the distal ends of the whiskers come in contact with Reward-platform. “Control” Gap-cross performance of bilaterally trained rats show that the contact with distal ends of whiskers is sufficient for the rats to judge the width of the gap and successfully cross it. Following experimental treatment, the rats with unilateral lesion (Group 2 and Group 3) or with unilateral whisker trimming (Group 4) or with trimming of whiskers from rows A, B and C contact the Reward-platform with the distal ends of the whiskers but do not jump across the gap. The difference between the “Control” and experimental conditions is that during “Control” performance the rats can acquire sensory information through intact whisker to barrel cortex pathways from both sides of the face whereas following experimental treatment the rats of Group 2, Group 3 and Group 4 have intact whisker to barrel cortex pathway from only one side of the face and in Group 5 the rats have only partial number of intact whiskers bilaterally. However if the gap-width is reduced by ~10%, the rats can jump across the gap. Gap-crossing at this reduced gap-width is also whisker-dependent since they cannot contact the Reward-platform with nose.

These results suggest the exact location on the shaft of the whisker that comes in contact with the Reward-platform could be relevant in sensory assessment of the gap-width. Szwed et al. (2006) have reported that touch neurons in the trigeminal ganglion fired more spikes when the objects were closer i.e., when proximal region of whisker shaft contacted the object. They also found that contact of the object with distal ends of the whisker activated very few touch cells compared to contact with proximal parts of the whisker. Neurons in the barrel cortex also show differences in the response magnitude when contact with the object occurs at different locations along the whisker shaft. Armstrong-James and Ebner (2003) observed reduction in the response magnitudes of neurons in the whisker-barrel cortex when the whiskers were stimulated from base to tip. They showed that response magnitude obtained when the base of the whisker (5 mm from pelage fur) was stimulated was three times more than when the whisker was stimulated at the middle and 10 times more than when the whisker was stimulated at the tip (within 2 mm of the free end). In the present study, although the rats contacted the Reward-platform with distal ends of their whisker, the total activity from bilateral pathway was sufficient to provide information about all aspects of the gap and hence, the rat can successfully cross the gap. In rats with unilateral lesions, the information of contact of Reward-platform is conveyed only through intact unilateral pathway, and the activity generated is insufficient for the rat to make correct judgment about the gap. With reduction of gap-width by ~10%, the position of the whisker shaft that makes contact with the Reward-platform is more proximal and hence could generate higher response magnitude, which perhaps is adequate for judgment of the gap.

#### Number of Whiskers That Contact the Reward-Platform

Similar to rats (Hutson and Masterton, 1986) mice also can learn to perform gap-crossing-task with single whisker (Celikel and Sakmann, 2007). However, the time taken by the mice to learn to perform the gap-crossing task successfully depended on number of whiskers used for learning the task. With single whisker the mice took longer to learn the task than with multiple whiskers. In our experiments we have observed that during performance of the gap-crossing task the rat actively scans the edge of the reward platform with its whiskers prior to jumping across the gap. Hutson and Masterton (1986) suggest that this active detection by whiskers requires whisker barrel cortex, whereas the whiskers could discriminate different frequencies of passive stimulation in the absence of barrel cortex.

The lengths of the whiskers on rats’ face are not uniform (Brecht et al., 1997; Haidarliu and Ahissar, 2001) and the distances between rostral tip of the nose to the whisker tip are also variable (Morita et al., 2011). Therefore, when crossing the maximum gap-width, it is likely that the rats contact the Reward-platform with fewer whiskers, but with whiskers from both side of the face. This bilateral input conveyed to the cortex is sufficient for the rats to make judgment about the gap. Whereas, in unilaterally lesioned animals, the contact of the Reward-platform is with half the number of whiskers and perhaps the information conveyed to the intact cortex is insufficient. However, when the gap-width is reduced by ~10%, it is possible that more number of whiskers can contact the Reward platform and therefore the lesioned rats can judge the gap to cross it successfully. This notion is supported by the study of Krupa et al. (2001). In their experiments, rats were trained to detect small differences in the sizes of apertures using all their whiskers on both sides of the face. They saw reduction in the detection of apertures when the whiskers were removed, with the degree of reduction correlating to the number of whiskers removed. Even when rats touched the aperture walls with 8–12 whiskers on each sides of the face there was ~25% reduction in discrimination of width of the apertures. In this study the rats in Group 5 showed deficit in performing the Gap-crossing even though they had two rows of large whiskers intact (D row and E row) on both sides of the face. Based on this result it can be argued that the inability of the animals to perform Gap-crossing is because of the reduction in the number of whiskers. Perhaps testing Gap-crossing task with increasing number of whiskers could give insight as to the amount sensory input required to perform the task optimally. Both Group 4 and Group 5 rats have normal cortex but reduced number of intact whiskers. Group 4 rats have all whiskers intact on one side of the face and the Group 5 rats have two rows of intact whiskers (D and E rows) on both sides of the face. At Gap*M* gap-width Group 4 animals had significant differences in the time spent on the Start-platform, number of attempts and time probing the gap with unilateral whiskers compared to control performance, while for the Group 5 animals there was no significant difference in these parameters. This implies that perhaps the bilateral activity between the areas corresponding to the D and E rows of whiskers has positive influence on the Gap-crossing behavior.

If the above-mentioned mechanisms are considered, it is possible that the level of activity or spike-trains in the trigeminal neurons and cortex could translate to performance of whisker-guided behavior. Yet in the Group 3 animals, with unilateral lesion and intact whiskers on both sides of the face, the activation of the trigeminal neurons bilaterally is not sufficient for successful Gap-crossing when the animal has to perform the task at maximum limit. While in Group 4 animals, with unilateral whiskers intact and no cortical lesion, and Group 5 animals, with partial number of intact whiskers bilaterally the neuronal response to contact Reward-platform also is not sufficient for successful Gap-crossing. The implication is that, manifestation of behavior in response to stimuli, at the highest limit of sensitivity, requires interhemispheric interactions of sensory input.

#### Interhemispheric Integration

In the current study, all the rats learned the Gap-crossing task using all the whiskers on both side of the face. Therefore, at any given instant both left and right cortices are activated by the inputs from the whiskers on both sides of the face. Bilateral integration and transfer of information occurs in the somatic sensory cortex (Ebner and Myers, 1962; Harris and Diamond, 2000; Shuler et al., 2002; Tommerdahl et al., 2006; Blankenburg et al., 2008) and both cortices are actively involved in coding for the stimuli (Ahissar et al., 2000). Both whisker trimming and SI lesions resulted in a reduction in performance of the behavioral task. Thus, bilateral activity is needed for the rats to discriminate sensory stimuli, such as the distance of the Reward-platform across a gap, and this information is required for making a decision about jumping across the gap.

Pidoux and Verley (1979) showed that activation of barrel cortex neurons by stimulation of ipsilateral whiskers was abolished when the contralateral barrel cortex was lesioned, supporting the conclusion that evoked responses to stimulation of the ipsilateral whiskers depends upon commissural inputs. Modulation of SI neurons by sensory inputs, via corpus callosum, has been reported by Shuler et al. (2001). They observed that responses of layer 5 neurons in SI to test stimuli was abolished following injury to homotopic contralateral cortex or also after cutting the corpus callosum. Reductions in spontaneous and evoked activity as well as impaired experience-dependent plasticity have been reported in the neurons of the intact barrel cortex following lesions of the homotopic region in the contralateral hemisphere (Rema and Ebner, 2003). The mechanism proposed to explain these lesion-induced effects is the low input activity to the intact barrel cortex through callosal inputs from the lesioned hemisphere (Li et al., 2005). Further studies by Li and Ebner (2006) showed that the response threshold of the neurons in the somatosensory thalamic relay nuclei is modulated by changes in the level of activity in the contralateral somatosensory cortex. In addition, the activity of thalamic VPM neurons can be modulated by the descending cortico-thalamic inputs (Temereanca and Simons, 2004; Li and Ebner, 2006; Andolina et al., 2007). Hence a unilateral lesion will affect not only the callosal inputs but also influence the corticothalamic and the thalamocortical activity in the contralesional cortex. Thus, we assume that there is an interhemispheric effect of injury on behavior, as a result of changes in the physiological properties of neurons, which is mediated through direct and indirect anatomical connectivity between the homotopic regions.

#### Alteration in the Spike Code and Threshold of Synaptic Modification

High speed video observations have shown that both whisker pads of rats usually move in synchrony, but can also, under some conditions, move asynchronously (Ahissar et al., 2000; Bermejo et al., 2002, 2005; Sachdev et al., 2002; Berg and Kleinfeld, 2003). Synchronous and asynchronous whisking assists in object perception (Hartmann et al., 2003; Towal and Hartmann, 2006, 2008). It has been reported that spatiotemporal features of a stimulus in the environment are coded by the latency and magnitude of the spike trains produced by neurons in response to whisker contact with the object (Ahissar et al., 1997, 2000; Ahissar and Arieli, 2001; von Hiemandahl et al., 2007). In animals with unilateral barrel cortex lesion the low neuronal activity in the intact cortex (Rema and Ebner, 2003) could affect the latency and magnitude of spike trains, and therefore, interfere in the coding of spatial and temporal aspects of the sensory stimuli thereby impeding behavior.

Neurons in the barrel cortex are sensitive to changes in the levels of input activity. Neurons of layers 2/3 have been shown to be the first to increase their stimulus-driven responses when sensory input is modified by trimming all but two whiskers and subsequently, this experience-dependent plastic change occurs in layer 4 and 5 neurons (Diamond et al., 1993; Armstrong-James et al., 1994; Rema and Ebner, 1999). A modeling study by Benusková et al., 1994 suggests that this type of experience-dependent plasticity operates by dynamic changes in the synaptic modification threshold. Discrimination of sensory inputs and subsequent processing of the sensory information for making decisions could involve active changes in this threshold. The synaptic modification threshold is dependent on the state of activity of the neurons (Benusková et al., 2001). The low spontaneous and evoked activities of neurons in the intact barrel cortex, contralateral to a focal lesion (Rema and Ebner, 2003) could alter the rate of synaptic modification needed for triggering behavioral output.

The synaptic modification threshold theory assumes that a specific quantity of synaptic activity is a prerequisite for the threshold to shift. Our hypothesis is that the amount of activity becomes crucial when performance of a behavior is based on very high threshold of sensitivity. Learning the Gap-crossing task with bilateral whisker inputs could result in altering the state of neuronal activity in each hemisphere. When Gap-crossing is being performed at the maximal distance, the input activity from the whiskers to the barrel cortex in each hemisphere is just below threshold. However, the callosal inputs bring additional activity from contralateral hemisphere. This combined activity is sufficient to raise it above the threshold for synaptic modification. Although, at present it is not certain whether threshold for synaptic modification is synonymous with onset of perception, it is possible that the ability of the rat to sense the platform and ability to judge before crossing the gap is directly influenced by the level of input activity. This would also explain the decrement seen in animals with unilateral lesions even though they used whiskers on both sides for the Gap-crossing task. The same mechanism could also explain reduction in Gap-crossing performance of normal rats with whiskers intact only on one side.

Human patients with focal stroke-like injuries of SI cortex show alterations in behavior and cognitive functions (Luft et al., 2004; Henry et al., 2006; Milders et al., 2008). A general feature of any injury to the cerebral cortex is loss of tissue at the injury site. Direct consequence of such tissue damage is modification in neuronal activity around the lesion site (Mattia et al., 2003; Machado et al., 2004; Ring et al., 2004; Jang et al., 2005). Spatio-temporal alterations in somatosensory evoked potential (SEP) and positron emission tomography (PET) of distant regions that have anatomical connections with the site of lesion injury have been reported in human patients (Nakashima et al., 1985; Forss et al., 1999; Mun-Bryce et al., 2004; Wu et al., 2004). Such deficits also referred as diaschisis (von Monakow, 1914), could be attributed to ongoing lesion-induced synaptic modifications in the intact regions as a result of the injury. This notion is supported by studies that show changes in the neuronal activity and behavior controlled by intact cortical regions reciprocally connected to the lesioned regions, in animal models (Rema and Ebner, 2003) and in children (Anderson et al., 2005; Tavano et al., 2009). Results of our study suggest that even though the major inputs to a cortical region remains intact, modification in anatomical or neurophysiological functions resulting from injury in a connected area could alter the sensitivity of the intact region and ultimately affect behavior.

## Author Contributions

VR designed the experiments. RC and VR performed the experiments and analyzed the data. VR wrote the manuscript with support from RC.

## Conflict of Interest Statement

The authors declare that the research was conducted in the absence of any commercial or financial relationships that could be construed as a potential conflict of interest. The reviewer YK and handling Editor declared their shared affiliation.
